# Testing the Role of Meander Cutoff in Promoting Gene Flow across a Riverine Barrier in Ground Skinks (*Scincella lateralis*)

**DOI:** 10.1371/journal.pone.0062812

**Published:** 2013-05-02

**Authors:** Nathan D. Jackson, Christopher C. Austin

**Affiliations:** Department of Biological Sciences, Museum of Natural Science, Louisiana State University, Baton Rouge, Louisiana, United States of America; Institut de Biologia Evolutiva - Universitat Pompeu Fabra, Spain

## Abstract

Despite considerable attention, the long-term impact of rivers on species diversification remains uncertain. Meander loop cutoff (MLC) is one river phenomenon that may compromise a river’s diversifying effects by passively transferring organisms from one side of the river to the other. However, the ability of MLC to promote gene flow across rivers has not been demonstrated empirically. Here, we test several predictions of MLC-mediated gene flow in populations of North American ground skinks (*Scincella lateralis*) separated by a well-established riverine barrier, the Mississippi River: 1) individuals collected from within meander cutoffs should be more closely related to individuals across the river than on the same side, 2) individuals within meander cutoffs should contain more immigrants than individuals away from meander cutoffs, 3) immigration rates estimated across the river should be highest in the direction of the cutoff event, and 4) the distribution of alleles native to one side of the river should be better predicted by the historical rather than current path of the river. To test these predictions we sampled 13 microsatellite loci and mitochondrial DNA from ground skinks collected near three ancient meander loops. These predictions were generally supported by genetic data, although support was stronger for mtDNA than for microsatellite data. Partial support for genetic divergence of samples within ancient meander loops also provides evidence for the MLC hypothesis. Although a role for MLC-mediated gene flow was supported here for ground skinks, the transient nature of river channels and morphologies may limit the long-term importance of MLC in stemming population divergence across major rivers.

## Introduction

Although much attention has been paid to understanding the role of large rivers in biogeography, their impact on species diversification is still controversial [1]–[6]. Major rivers undoubtedly impede dispersal for many terrestrial organisms, but these rivers may not be sufficiently impenetrable or long-lived to commonly facilitate speciation [1], [7]. For example, a river that is wide and fast-flowing near its mouth may be circumnavigated around its headwaters. Furthermore, a river that is a major dispersal barrier today may not have been so in the past (e.g., during the Pleistocene) when conditions were cooler and more arid.

**Figure 1:**
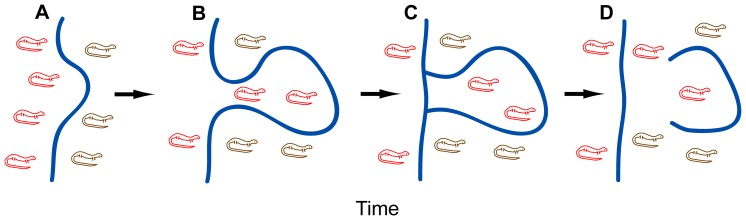
How meander loop cutoff can facilitate gene flow across a riverine barrier. **A**, Isolated populations on opposite sides of a river may be genetically distinct (represented by red and brown lizards). **B**, Increasingly large meander loops may form due to erosion and re-deposition of channel sediments. **C**, Meander loops may expand to the point of being cut off at the neck by the forging of a more direct channel. Individuals living within the meander loop are passively transferred across the river. **D**, Ancient meander loops recede from the main river channel, forming oxbow lakes. Alleles transferred across the river are released into a novel population, resulting in unidirectional gene flow.

Meander loop cutoff (MLC) is an additional factor often cited to explain why rivers are poor long-term dispersal barriers [1], [3], [5], [8]–[10]. MLC is a recurrent phenomenon whereby widening river meander loops become severed from the main river channel due to a natural process of channel straightening [11], [12]. Aside from producing oxbow lakes, this process can result in the passive transfer of habitat (along with all the organisms that live there) from one side of the river to the other (Figure 1; [1], [3], [8]). For a single meander cutoff event, foreign alleles passively transferred by the cutoff may have no long-term population genetic effect. However, if MLC rates are sufficiently high, gene flow may overwhelm the differentiating effects of mutation, drift, and selection acting on opposite sides of the river. Although often cited as a problem for the riverine barrier hypothesis, passive transport due to MLC has yet to be empirically demonstrated, likely due in part to the logistical challenge of collecting large samples of individuals along a series of these ancient meander loops.

In this study, we investigate the role of MLC in facilitating gene flow between lineages of a common lizard species that are separated by a well-known riverine barrier, the Mississippi River. Ground skinks, *Scincella lateralis*, are highly abundant [13], [14] and vagile [15], [16] lizards endemic to mesic habitat throughout the southeastern United States. Broad scale genetic research on *S. lateralis* has revealed several genetic discontinuities that align with major southeastern rivers, suggesting that these rivers have historically isolated populations [17]. Despite this history of divergence, phylogeographic evidence suggests that some gene flow is occurring across rivers and thus that these riverine barriers may not be complete [18]. This is particularly true of the Mississippi River, one of the most cited dispersal barriers in the southeastern US [19]. This river divides genetically distinct populations within a variety of taxa (reviewed in [19], [20]) and generally coincides with the boundaries of two *S. lateralis* mtDNA lineages (separated by an average uncorrected divergence of 6.2% [17]). Despite this divergence in *S. lateralis*, there is some lineage overlap at sites close to the river, particularly near the river delta in southern Louisiana [17]. The boundary between two populations inferred using eight nuclear loci is characterized by even more extensive overlap than observed for mtDNA, and an isolation-with-migration model fits the multilocus data from these two populations better than an isolation-only model [18]. Thus, the Mississippi River appears to be acting as a “leaky barrier” for ground skink populations.

**Figure 2:**
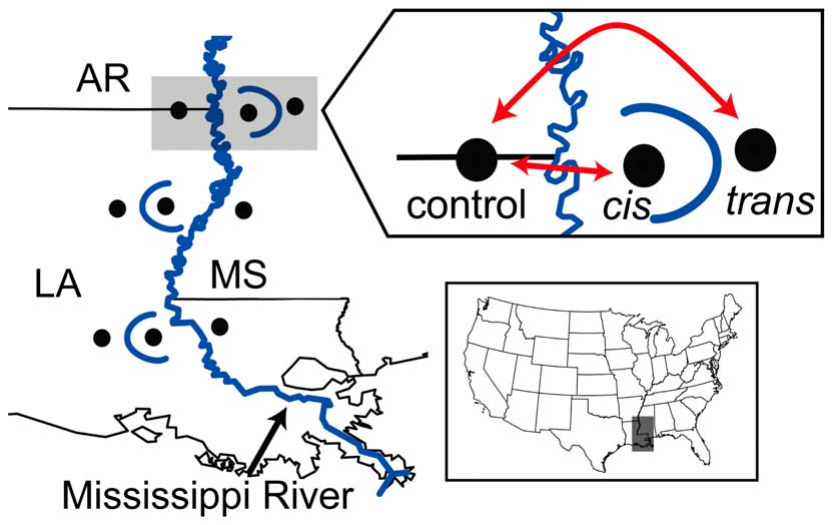
Study design. Triplets of sites (*cis*, *trans*, and control) were sampled for each of three oxbow lakes along the Lower Mississippi River to test for patterns of gene flow and divergence expected under meander loop cutoff-mediated dispersal. Under MLC, we predict the following: 1) *cis* should be more closely related to control than to *trans*; 2) *cis* should contain more individuals that genetically “belong” on the opposite side of the river than the control; 3) immigration rate estimates should be higher control → *cis* than *cis* → control; 4) the pre-cutoff river channel (where *cis* and control are on the same side of the river) should predict the distribution of private alleles (i.e., alleles native to one side of the river) better than the post-cutoff river channel (where *cis* and control are on opposite sides of the river).

Meander loop cutoff is one mechanism by which gene flow across the river may be maintained. The meander belt of the Lower Mississippi River (south of Cairo, Illinois) has been estimated to historically generate 13–15 oxbow lakes per century [21]. Thus, MLC may be sufficiently common to provide an effective “revolving door” by which passive dispersal can take place across an otherwise impermeable barrier. MLC-mediated dispersal is predicted to leave behind a distinct population genetic signature, if caught soon enough after the cutoff event (Figure 2). First, immigrants should be more closely related to individuals across the river than on the same side. Second, more immigrants should be observed at sites that are near oxbow lakes than at sites that are not. Third, population migration rates across a river estimated near an oxbow lake are expected to be asymmetrical, with immigration occurring in the direction of the cutoff event. Finally, the distribution of alleles that are native to one side of the river should be better predicted by the historical (pre-cutoff) river channel than the current (post-cutoff) channel. In this study, we investigated whether MLC has contributed to cross-river gene flow in ground skinks by testing the predictions of MLC-mediated dispersal. To do this, we analyze mtDNA and microsatellite data from individuals collected near three oxbow lakes along the Lower Mississippi River.

## Materials and Methods

### Sampling Design

**Figure 3:**
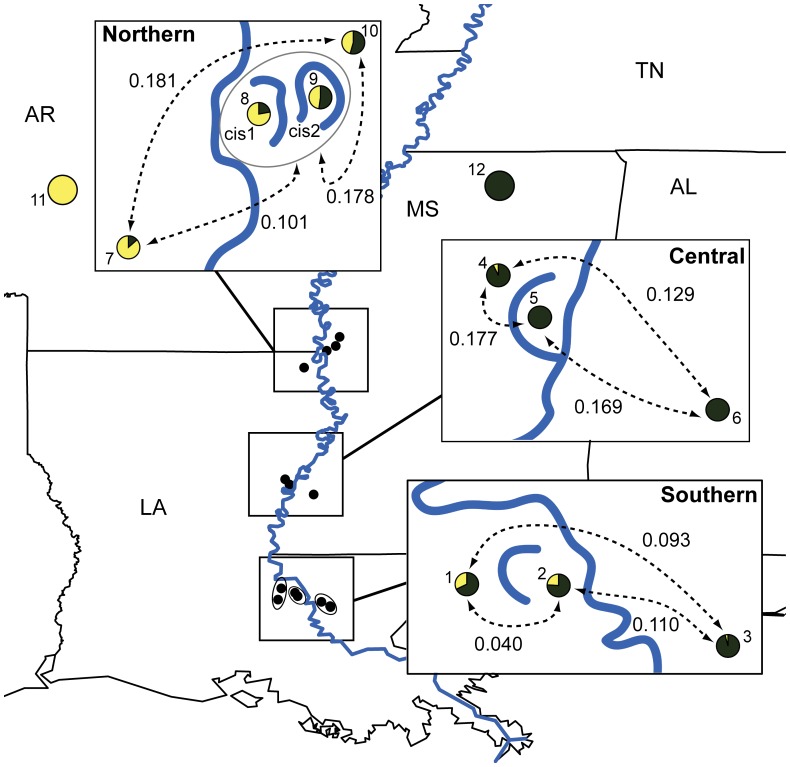
Distribution of mtDNA haplogroups and microsatellite divergence in relation to three Mississippi River oxbow lakes. Circles refer to sample sites and numbers correspond to localities described in Table S1. Two “pure” sites (11 and 12) were sampled away from the river. For each site, circle fill is proportional to percent membership of samples in two cyt*b* haplogroups (yellow = western haplogroup, green = eastern haplogroup). Dotted lines between sites indicate *D_est_* values for site pairs calculated from microsatellite data. For the southern oxbow samples, circles around sampling sites indicate multiple locations that were treated as a single site. In the north, two *cis* sites were collected (*cis*2 and *cis*2) and combined for some analyses to increase sample size.

We collected tissue from 260 lizards from 15 sites located along both sides of the Lower Mississippi River (Table S1). Thirteen of the sampling sites are located near three oxbow lakes (Figure 3): Lake Washington (LW) in Washington County, Mississippi (formed ∼635 ybp), Lake St. John (LSJ) in Concordia Parish, Louisiana (formed ∼535 ybp), and False River (FR) in Pointe Coupee Parish, Louisiana (formed ∼300 ybp; dates are approximate and based on [21], [22]). We refer to these three sampling locations as northern, central, and southern sites, respectively.

Oxbow selection was based on 1) geographical equidistance among sites, 2) apparent availability of suitable habitat for *S. lateralis* at *cis* sites as inferred from inspecting current aerial photographs, and 3) the lack of evidence for ancient oxbow lakes on the side of the Mississippi River opposite from *cis* and *trans* sites such that appropriate control sites could be sampled. For each oxbow, we sampled sites inside (*cis*) and outside (*trans*) the ancient meander loop (Figure 2). Ideally, control sites would be sampled in locations along the river without a history of MLC to compare with sites sampled near oxbow lakes. However, because of the high rate of MLC along the Mississippi River, no site near the river is very far from a current or past oxbow lake [22]. Thus, we sampled sites across the river from each of the three oxbows to use as paired controls; the MLC transfer hypothesis predicts that the relative number of migrants at an oxbow site should be higher than at its paired control site across the river (Figure 2). In selecting control sites, we ignored recent cutoffs that still retain residual connection to the main river because these have not yet likely released immigrants into the population [21]. Finally, we also sampled two sites far removed from the Mississippi River (Montgomery Co., AR and Benton Co., MS) referred to as “pure” sites which are assumed (based on previous mtDNA and nuclear sequence data [17], [18]) to exist away from the region of contact between groups separated by the river (Figure 3).

Due to the limited number of samples obtained just *cis* of the northern oxbow (Lake Washington), Swan Lake, a prehistoric oxbow lake (formed ∼1,350 ybp) that has since undergone transformation into a low-lying swamp and which lies a few kilometers east of Lake Washington was also sampled (Figure 3). We also sampled multiple locations per site near the southern oxbow. Where multiple *cis*, *trans,* and control sampling locations were used at a single oxbow, these were combined for most analyses such that 20–29 samples were available for each *cis, trans,* and control site (Figure 3). No significant differentiation was detected among sampling locations within sites using analysis of molecular variance (AMOVA; P>0.05; [23]).

### Ethics Statement

All lizards were handled in accordance with guidelines compiled by the Herpetological Animal Care and Use Committee (HACC) of the American Society of Ichthyologists and Herpetologists (found at http://www.asih.org/files/hacc-final.pdf). This study was carried out under an approved Louisiana State University IACUC protocol (#A3612–01).

### Genetic Methods

We extracted genomic DNA from liver or tail tissue preserved in 95% ethanol using salt-extraction (Fetzner 1999) or a Qiagen DNeasy extraction kit (Qiagen, Valencia, CA), respectively. For all samples we genotyped 13 microsatellite loci developed previously for *S. lateralis* [24] and sequenced 809 base pairs of the cytochrome *b* (cyt*b*) mitochondrial gene using the primers L147241 [25] and CCAM504R [17]. We carried out polymerase chain reaction (PCR) for mtDNA and microsatellite loci as described previously [17], [26]. For microsatellites, PCR products were electrophoresed on a 3100 Genetic Analyzer and scored using Genemapper v3.7 (Applied Biosystems) against a Naurox size standard [27]. For cyt*b*, amplicons were purified by combining 5 µl PCR product with 0.25 µl Exonuclease I (20 units/µl), 0.25 µl of Antarctic phosphatase (5 units/µl), 0.25 µl 10× buffer (50 mM Bis-Tris Propane/HCl, 1 mM MgCl_2_, 0.1 mM ZnCl_2_), and 4.25 µl purified water, followed by incubation for 20 min at 37°C and 15 min at 80°C. Cycle-sequencing was carried out for each amplicon using a BigDye Terminator cycle-sequencing kit version 3.1 (Applied Biosystems, Foster City, CA). After sequences were cleaned using Sephadex, they were electrophoresed on a 3100 Genetic Analyzer (Applied Biosystems).

We calculated the error rate in microsatellite genotyping by repeat-genotyping (both randomly and non-randomly) a subset of samples. We first randomly selected 16 samples for each locus (∼6% of the total dataset) to be re-genotyped. Secondly, we also purposefully re-genotyped 76 samples that exhibited low peak intensity upon initial genotyping. Reaction error rate was calculated by dividing the number of mismatched genotypes by the total number of re-genotyped samples [28], [29].

Cyt*b* sequences new to this study have been deposited in GenBank under accession numbers KC762326 - KC762565; 21 cyt*b* sequences used here have been published previously [17]. For microsatellite genotypes and corresponding GenBank accession numbers, see Dataset S1 in the Supporting Information.

### Population Structure

For all loci and sites we calculated allele number, Nei’s unbiased estimate of gene diversity [30], and Weir and Cockerham’s *F_IS_* index [31] using FSTAT ver2.9.3.2 [32]. We calculated allelic richness using HP-RARE [33] which uses rarefaction to adjust for differences in sample size. Proportions of observed heterozygosity were calculated using Cervus ver3.0.3 (Marshall et al. 1998) and exact tests for Hardy-Weinberg equilibrium (HWE) and linkage disequilibrium among loci were carried out using GENEPOP ver3.4 [34].

We investigated genetic structure among sampling sites to test two predictions of the MLC hypothesis. First, if MLC is responsible for gene flow across the Mississippi River, the number of individuals sampled on the “wrong” side of the river (i.e., with a genetic profile different from those in the pure site on the same side of the river) should be higher on the oxbow side of the river than on the control side. For cyt*b*, we investigated this by assigning all samples to one of two mtDNA clades previously described in the region [17]. This was done using a cyt*b* gene tree which we inferred by carrying out a full maximum likelihood (ML) search with 1000 rapid bootstrap pseudoreplicates using RAxML ver7.2.6 [35]. For the microsatellite loci, we estimated river-based population assignments using the Bayesian clustering algorithm in the program STRUCTURE ver2.2.3 [36]. We ran the program for each of the three latitudes (northern, central, and southern groups of sites) separately (including the two pure sites in each analysis) under the assumption of *K = *2 populations. We also estimated the true *K* for each latitude by comparing log-likelihoods of the data under a series of models assuming different *K* numbers of populations (from *K* = 1 to *K* = 5). We ran the program ten times for each model for at least 1 million generations (with an additional burnin of 500,000).

Secondly, if lizards were recently transported across the river due to MLC at the three oxbow sites, then *S. lateralis* collected from *cis* sites should have a closer genetic affinity to *S. lateralis* from across the river (control sites) than from the same side (*trans* sites). To investigate this prediction using microsatellite loci, we calculated divergence metrics *D_est_* [37], Wright’s *F_ST_*, and *G’_ST_* [38] for population pairs using AMOVA in Arlequin ver3.1.1 [39]. Although *F_ST_* can underestimate divergence when rapidly mutating markers such as microsatellites are assayed [40], [41], we selected *F_ST_* over the alternative metric *R_ST_* (which takes into account a stepwise mutation model; [42]) due to the observation of superior overall performance of the former in studies which, like ours, involve limited sampling and recent divergence [43]–[45]. *D_est_* and *G’_ST_* were calculated using SMOGD ver1.2.5 [46]. These metrics are independent of the degree of genetic diversity within a dataset and are thus particularly suitable for microsatellite loci [47].

### Migration Rates

If the recent ancestors of *cis* individuals were passively transferred across the Mississippi River due to MLC, estimates of gene flow should be asymmetrical, trending higher in the direction of the oxbow. Detection of symmetrical gene flow would support non-MLC causes of dispersal. To test this prediction, we used the program BIMr [48] to estimate recent immigration rates among sampling sites. This program uses a Bayesian assignment test algorithm that has been shown to effectively estimate recent migration rates even when they are relatively high [48], as is expected here. We first estimated rates among pure sites and river sites (*cis*, *trans*, and control) at northern, central, and southern localities separately. For each analysis, we ran a Markov chain for 50,000 burnin samples, followed by 50,000 samples collected using a thinning interval of 50. Convergence of the Markov chain was assessed by repeating independent analyses six times.

As a comparison to recent estimates of gene flow, we also estimated the effective number of migrants per generation (*Nm*) among pairwise populations using the private alleles method implemented in GENEPOP ver3.4 [34], which assumes an infinite island model of migration and quasi-equilibrium within populations [49].

### Distribution of Private Alleles

We next investigated the distribution of private alleles (defined here as alleles found in one pure site but not the other) along the Mississippi River. If individuals have been passively transported across the river due to MLC, then the historical (pre-MLC) river course should better predict the distribution of private alleles than the current (post-MLC) river course. Because there are a large number of private alleles inherent in microsatellite data, we isolated those alleles that we predicted would be the most informative. There were 79 private alleles from the 13 loci, but only 16 were common enough within their respective pure sites (had a ≥15% prevalence) that we considered them sufficiently diagnostic of either pure site. Six of those 16 alleles were both a) located on independent loci and b) common enough throughout the whole dataset (≥10% frequency) to consider in our analysis.

Using these six microsatellite alleles along with cyt*b* (scored as bi-allelic), we carried out two logistic regression analyses to test whether the proportion of individuals at a site in possession of a particular private allele (the response variable) is better explained by that site’s location relative to the historical or current river channel. Specifically, the predictor in each model was a binary variable describing whether a site is on the same side of the river (“native”) or across the river (“non-native”) from a particular allele’s pure site of origin. The model was run separately for predictors scored according to the historical and current paths of the river. In the historical model, *cis* sites were grouped with control sites, whereas in the current model, *cis* sites grouped with *trans* sites. We then compared the relative fit of the historical and current models using AIC. We also repeated the analysis with and without cyt*b.* All models incorporated latitude (northern, central, and southern) and allele (seven groups) as random effects based on significant improvement in model fit when included (ΔAIC = 48). All analyses were carried out using R [50].

## Results

### Genetic Methods

The genotype error rate was 0.0096 errors per reaction for the set of samples randomly re-genotyped. These errors were due to mistakes in scoring rather than to allelic dropout. No genotype mismatches were observed for the 76 samples non-randomly re-genotyped, which, when combined with the set of randomly re-genotyped reactions, yields a total genotype error rate of 0.0070 errors per reaction.

### Population Structure

Average *F_IS_* values for these sites were all positive, ranging from 0.11 to 0.26, indicating a pervasiveness of heterozygote deficiency. Pairwise tests of significance for this deviation from Hardy-Weinberg equilibrium across loci and populations were significant in 42 out 143 total tests, involving eight of the 13 loci and all sites (after a Bonferroni correction for multiple tests). Given that we have sampled within the putative contact zone between two genetically distinct populations, a Wahlund effect produced by the co-occurrence of unique gene pools within sampling sites is a likely reason for the observed disequilibrium. This is supported by the observation that the western pure site, the site farthest from the river, exhibits the least amount of disequilibrium (Table 1). Null alleles [51] due to mutations in flanking DNA regions are not likely the major cause of the observed disequilibrium given that all loci were developed specifically for *S. lateralis* and that the majority of loci used exhibit some degree of heterozygote deficiency, a pattern more parsimoniously attributed to one evolutionary process (genuine disequilibrium) than to many (artifactual disequilibrium; [52]). *F_IS_* at one locus (P2G08) was significantly positive in the western pure site, and as a precaution, was excluded from further analysis. No significant genotypic disequilibrium was detected among loci after a Bonferroni correction and average genetic diversity was quite high (Table 1).

**Table 1:** Average diversity indexes across 13 microsatellite loci for sites used in this study.

Locality	Site	Loc #	*N*	*A_N_*	*A_R_*	A_R_*	*H_E_*	*H_0_*	*F_IS_*
North	*cis*	8–9	30	12.0	8.48	6.50	0.839	0.676	0.213
North	*trans*	10	22	10.5	8.04	6.21	0.803	0.691	0.177
North	control	7	29	12.2	8.74	6.64	0.848	0.678	0.215
Central	*cis*	5	28	11.8	8.19	6.35	0.818	0.708	0.137
Central	*trans*	4	28	11.6	8.48	6.55	0.842	0.667	0.225
Central	control	6	21	11.5	8.71	6.69	0.847	0.703	0.178
South	*cis*	2	25	11.5	8.54	6.52	0.847	0.702	0.191
South	*trans*	1	25	11.8	8.59	6.49	0.842	0.667	0.207
South	control	3	25	12.9	9.15	6.88	0.866	0.668	0.239
West	pure	11	15	9.3	8.02	6.16	0.836	0.754	0.113
East	pure	12	12	8.7	8.03	6.11	0.811	0.611	0.262

Loc #: locality numbers corresponding to those in Figure 3 and Table S1; *N* = sample size; *A_N_* = allele number; *A_R_* = allelic richness; A_R_* = allelic richness adjusted for unequal sample size; *H_E_* = Nei’s unbiased gene diversity (expected heterozygosity); *H_0_* = observed heterozygosity.

For the cyt*b* dataset, there were 172 variable sites and 119 unique haplotypes. Two inferred lineages were separated by an average uncorrected pairwise distance of 6% (see the ML tree in Figure S1). Eastern and western haplogroups each contained all individuals from eastern and western pure sites, respectively. To different extents, predominantly western clade sites transitioned to predominantly eastern clade sites from west to east across the river. For all three latitudes, control sites contained a lower proportion of immigrant haplotypes than *cis* and *trans* sites (Figure 3), as expected if MLC helped facilitate gene flow across the river.

**Figure 4:**
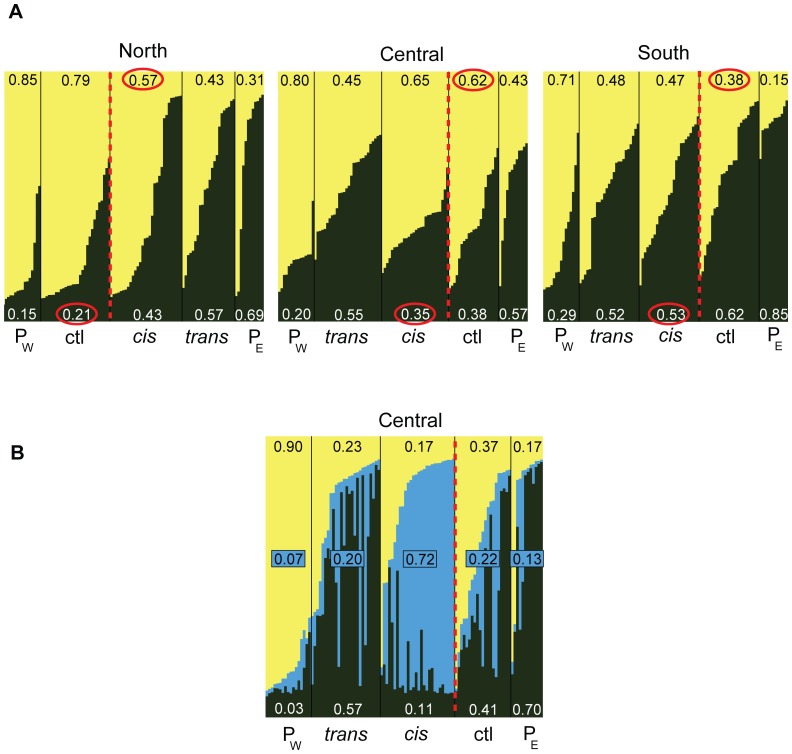
Distribution of microsatellite ancestry based on cluster analysis. **A**. Population assignments shown were inferred under the assumption of *K = *2 (**A**) or *K = *3 populations (**B**; central oxbow only). Separate analyses were carried out for northern, central, and southern sites (with the two pure sites included each time). Vertical bars represent individuals, each colored in proportion to probability of assignment to one of two (or three) populations. Bars are first clustered by latitude, then sorted east to west by site (separated by black bars), and finally sorted by probability of assignment to populations. Total proportion of inferred ancestry is given for each population (color-coded) at each site. P_W_ and P_E_ = western and eastern pure sites, respectively. Dotted red line indicates the current location of the Mississippi River. More immigrant ancestry was inferred in *cis* sites relative to control sites (comparisons are circled in red) for northern and central oxbows (as expected under MLC). The opposite pattern was inferred for the southern oxbow.

For the microsatellite loci, when *K* = 2 was assumed for the three datasets, samples within pure sites tended to be less mixed than samples near the river (Figure 4A). For the northern and southern localities, control sites contained a lower proportion of inferred migrant ancestry than *cis* or *trans* sites, a pattern consistent with expectations under MLC. When *K* was allowed to vary, *K* = 2 produced the highest log-likelihoods in the north (and the highest *ΔK*; [53]), whereas log-likelihoods peaked at *K* = 1 for the southern localities. The central pattern was more puzzling in that the *trans* site west of the river was dominated by eastern ancestry and the control site east of the river was more dominated by western ancestry. When *K* = 3 was assumed (which yielded the highest *ΔK* for the central localities), *cis* samples were predominately assigned to their own population (Figure 4B).

Genetic divergence among sites was significant for northern and central (but not southern) samples, regardless the divergence metric used (Table S2; *D_est_* values are given in Figure 3). In the north, when the Lake Washington and Swan Lake *cis* sites (called *cis1* and *cis2*, respectively) were combined, genetic differentiation was lowest between *cis* and control sites (Figure 3), as expected if gene flow is the result of a recent MLC event (regardless whether using *F_ST_*, *G’_ST_*, and *D_est_*). When the two *cis* sites were treated separately, *cis1* was the most genetically distinct site in all comparisons (Table S2). At the central and southern localities, *trans*-control and *cis-trans* comparisons exhibited the lowest genetic differentiation, respectively.

### Migration Rates

**Figure 5:**
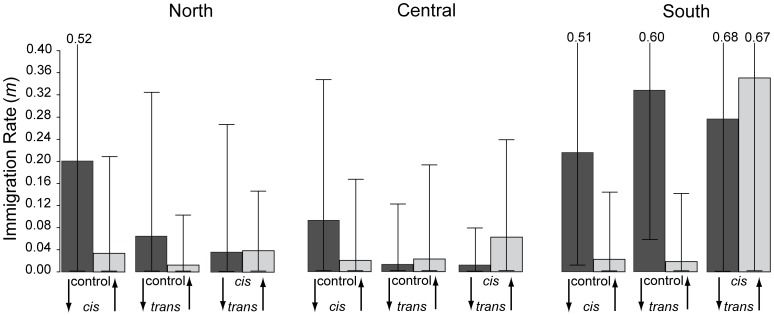
Asymmetrical immigration rate estimates from BIMr for pairs of sites near three Mississippi River oxbows. Ninety-five percent highest posterior density intervals are shown around each mean estimate (upper bound is provided numerically if extremely high).

Asymmetrical immigration rates estimated using BIMr were consistent among the six independent runs suggesting that convergence of the Markov chain had been reached. For all three latitudes, mean immigration rates were higher from control → *cis* sites than vice versa, consistent with the prediction that the ancestors of *cis* samples predominantly originated from across the river (Figure 5). Nevertheless, highest posterior density intervals (HPDI) around mean estimates were generally wide, and only in the southern oxbow analysis did the mean of one estimate not fall within the HPDI of the other. Rates also trended higher from control → *trans* than *trans* → control in the north and south, perhaps indicative of post-MLC movement of immigrant alleles beyond *cis* sites. *Nm* estimates trended higher directly across the river (control ↔ *cis*) than between sites on the same side (*cis* ↔ *trans*), as predicted under MLC (Table S3).

### Distribution of Private Alleles

**Figure 6:**
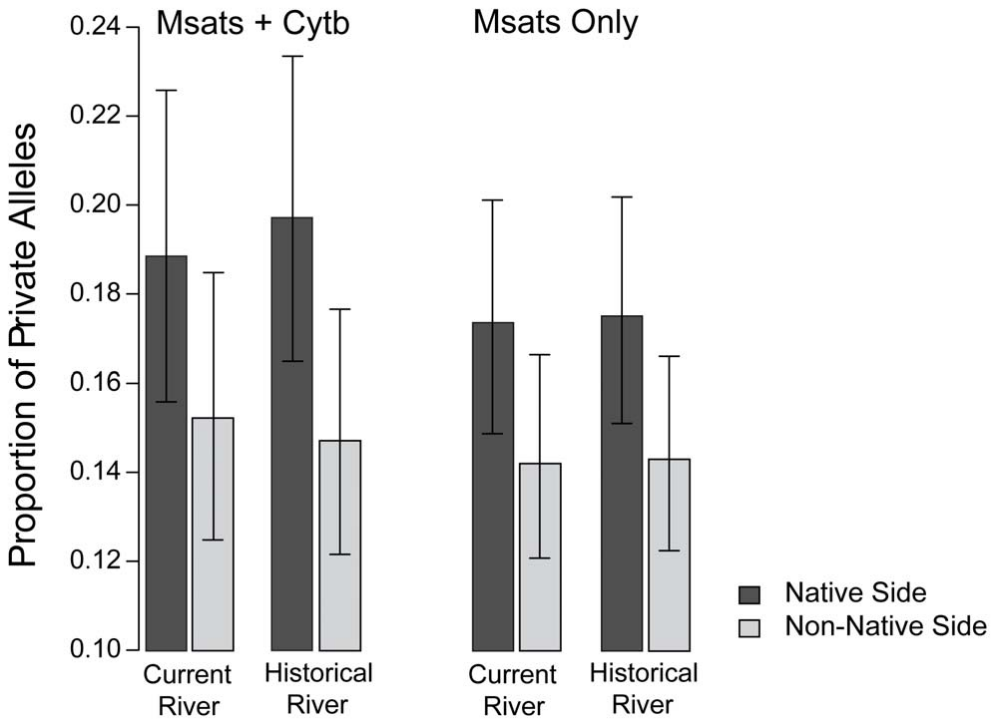
The distribution of private alleles as predicted by the current versus historical Mississippi River channel. The proportion of private alleles (averaged across six microsatellite alleles and two cyt*b* haplogroups unique to one pure site) was higher on their native side of the river than on their non-native side, based on logistic regression (p-values<0.05 in all cases). Results are shown including and excluding cyt*b*. For the combined data, the historical (pre-cutoff) river channel predicts the distribution of private alleles better than the current (post-cutoff) channel. This difference largely disappears when only microsatellite loci are analyzed. Standard error bars are shown for each estimate.

Regardless whether the Mississippi River was defined by its current or historical path, alleles private to one of the two pure sites were more common on their native side of the river than on their non-native side. Specifically, private alleles were 1.29 times (SE = 1.17–1.43, z = 2.58, P = 0.01) more common on their native versus non-native side of the river when the pre-MLC river channel was assumed, and 1.42 times (SE = 1.30–1.57, z = 3.60, P<0.001) more common when the post-MLC river channel was assumed, indicating restricted gene flow across the river. Furthermore, model fit was better when the river was defined by its historical channel rather than its current channel (ΔAIC = 6.3; Figure 6), lending modest support to the hypothesis that *cis* sites were recently positioned on the opposite side of the river. This improvement in model fit with historical classification was reduced however when cyt*b* was removed from the dataset (ΔAIC = 0.4).

## Discussion

### Meander Loop Cutoff-Mediated Gene Flow

Previous work on *S. lateralis* has provided evidence for genetic fragmentation across the Mississippi River at multiple loci, despite ongoing gene flow between populations [17], [18]. In this study, we have sampled several sites within the Lower Mississippi River Valley to investigate one possible mechanism–MLC-mediated passive transfer–by which gene flow across the river may be facilitated. In general, the distribution of genetic variation along the river supports predictions of the MLC hypothesis (Table 2). First, the distribution of two cyt*b* haplogroups in relation to the three oxbows sampled is generally consistent with oxbow transfer, which predicts a higher proportion of migrant genotypes at oxbow sites relative to control sites (found at all three sites). Moreover, at northern and central localities (but not southern), individuals within the ancient meander loop (*cis* sites) are genetically more similar to individuals across the river (control sites) than to individuals on the same side (*trans* sites; Figure 3). Secondly, the distribution of ancestry inferred for microsatellite loci using Structure are also generally consistent with these expectations for northern and southern oxbows, but not for the central oxbow (Figure 4A). Third, the historical (pre-cutoff) Mississippi River channel better predicts the distribution of two cyt*b* haplogroups than the current (post-cutoff) river channel, supporting MLC-mediated dispersal. A similar, although much weaker, result was observed for microsatellite private alleles. Fourth, immigration rate estimates are asymmetrical in the direction expected under the MLC hypothesis for all three oxbows (Figure 5). The directional bias in migration is particularly evident in the southern locality, possibly resulting from a more recent transfer event. Migration estimates using the private allele method also trend higher across the river than along the same side (Table S3). These genetic signatures of MLC-mediated gene flow are most consistently observed for the northern-most oxbow, perhaps in part due to its greater distance from the river delta, whose relative dynamism since the Pleistocene may have reduced across-river divergence (discussed further below).

**Table 2:** Summary of predictions for MLC-mediated gene flow and whether each prediction trended in the expected (Yes) or unexpected (No) direction.

Prediction	Loci	North	Central	South
*cis* are more genetically similar to the control than to *trans*	cyt*b*	**Yes**	**Yes**	No
	msats	**Yes**	**Yes**/No^b^	No
Number of individuals on the “wrong” side of the river are higher in *cis* than the control	cyt*b*	**Yes**	**Yes**	**Yes**
	msats	**Yes**	No	**Yes**
Asymmetrical immigration rate estimates are higher control → *cis* than vice versa	msats	**Yes**	**Yes**	**Yes**
Asymmetrical immigration rate estimates are higher control → *trans* than vice versa	msats	**Yes**	No	**Yes**
*Nm* is higher control ↔ *cis* than *cis* ↔ *trans*	msats	**Yes**	**Yes**	**Yes**
The historical river predicts the distribution of private alleles better than the current river^a^	msats+cyt*b*	**Yes**	**Yes**	**Yes**
Genetic divergence of *cis* sites is evident	msats	**Yes**	**Yes**	No

a All three rivers were tested simultaneously in this analysis.

b This prediction was ambiguous among the three metrics used.

Finally, one unanticipated pattern that also supports MLC-mediated dispersal is divergence of sites within ancient meander loops (*cis* sites). The best supported structure for the central locality invokes a third cluster that is predominately assigned to the *cis* site (Figure 4B). This suggests that samples on the *cis* side of the oxbow have been somewhat isolated (also supported by elevated *D_est_*, *F_ST_*, and *G’_ST_* values; Table S2), which makes sense in light of the central oxbow lake being very close to the Mississippi River, leaving a single small passageway from which transferred individuals can disperse into non-transferred habitat (Figure 3). Similarly, the *cis1* site is the most divergent site at the northern locality, with pairwise site comparisons involving *cis1* also consistently yielding the highest divergence values (Table S2). Once a meander loop has been severed from the river, habitat within the loop can remain isolated for long periods given that the tips of oxbows can require centuries to recede away from the main river channel [21]. Small populations trapped for generations between the current and historical river channels can quickly diverge due to genetic drift. Thus divergence of *cis* sites further suggests that the ancestors of *cis* samples were passively transported. If the ancestors of individuals in *cis* sites instead invaded *cis* habitat after the cutoff event, migration into and out of the site would likely have been free enough to impede divergence. That *cis* divergence is not observed for the southern oxbow may result from a more recent cutoff event and a separation from the river that was artificially hastened by anthropogenic levee construction [21], facilitating free genetic exchange with the native population early on.

### Isolation and Gene Flow across the Mississippi River

Intensive sampling of mtDNA and microsatellite loci near the Mississippi River has revealed that gene flow rates across the river are remarkably high. The high level of dispersal inferred across the river is surprising given the apparent importance of rivers as isolating barriers for *S. lateralis* throughout their range [17]. Furthermore, multiple lineages within co-distributed taxa tend to show high fidelity to the expected side of the river, with dispersal being rare, even when sampled near the delta (reviewed in [19], [20]). Pyron and Burbrink [20] calculated the probability of dispersal across the Mississippi River to be 0.057 using the distribution of lineages from nine phylogeographic studies. However, most of these studies have not focused on dense sampling near the river and may thus be insufficient guides to the extent of migration occurring currently across this riverine barrier in spite of divergence across it. Studies that have sampled more intensively near the river have found higher rates of population overlap [54], [55]. Furthermore, several terrestrial taxa have shown little to no genetic signature of the Mississippi River [56]–[61], suggesting that dispersal across the river is often sufficiently high to overwhelm divergence.

Genetic divergence in the face of ongoing gene flow has been reported, although usually in conjunction with selection [62]–[65] or small population sizes [66], [67]. These forces however do not appear to be major contributors to the maintenance of population divergence in *S. lateralis*. Large effective population sizes have previously been estimated for *S. lateralis* populations [18]. Also, current and past patterns of vegetation and climate suggest that the Mississippi River Valley does not delineate an ecological transition [68], [69]. Additionally, previous analysis of *S. lateralis* sampled at the Mississippi River Delta, where abundant channel switching has likely weakened its barrier effect [70], reveals a correspondingly wider contact zone between divergent groups [18], in accordance with neutral expectations [71], [72].

Divergence in the face of gene flow between parapatric populations can also result when divergence and gene flow are decoupled in time. Divergence may largely occur in allopatry, followed by (cycles of) post-divergence gene flow. This could explain the non-equilibrium conditions inferred at the region of contact and high contemporary estimates of gene flow between populations. Throughout cycles of waning Plio-Pleistocene glaciation, significant alluviation along a wide network of braided streams expanded the Mississippi River Valley, possibly bolstering the isolating force of the river [73], [74]. The single meandering channel presently bisecting the valley may comprise the latest in a cyclical series of such channels where impermeability of the river has been relaxed, potentially due in part to channel migration inherent in the meander belt system.

Mitochondrial and microsatellite loci differ in their patterns of divergence across the river. The contact zone for microsatellite populations is much wider than for mtDNA lineages, incorporating sites distant from the river (pure sites). Furthermore, the degree of divergence is much lower among inferred microsatellite populations than among mtDNA lineages. One possible explanation for this difference among markers is increased homoplasy in microsatellite loci due to their higher mutation rates, which could partly erase the signature of ancient divergence [75], [76]. This is particularly expected to occur when effective population size is high [75], as is found in *S. lateralis*. Secondly, despite high mutation rates, divergence may still proceed more slowly in microsatellites than in mtDNA due to more gradual sorting of ancestral alleles in nuclear loci. This is because nuclear loci have a four-fold larger effective population size than mtDNA. Third, male-biased dispersal could increase the distance over which immigrant alleles move away from the river in nuclear DNA relative to mtDNA. This is plausible in *S. lateralis* where males have been shown to use three and a half times the home range of females [15].

### Limitations on the Influence of Meander Loop Cutoff

Although genetic patterns broadly correspond to those predicted under MLC-mediated dispersal, gene flow detected across the river may have also been aided by other types of river channel movement. For example, the Mississippi River meander belt has shifted several times since the Last Glacial Maximum (LGM). Throughout much of the Holocene, flow of the Mississippi River was shared among two or more meander belts, a general rule to which the single wide channel presently occupied by the river is an exception [74], [77]. One such ancient meander belt, lying in the Yazoo Basin ∼70 km east of the current channel near Lake Washington (the northern locality), was likely an important distributary channel to the main course until as recently as 3,000 years before present (ybp) and likely contained the main trunk of the river as recently as 8,000 ybp [74], [77]. In addition, a channel within Tensas Basin (∼15 km west of the central oxbow, Lake St. John) is thought to have carried a significant proportion of the Mississippi River Valley flow approximately 3,000 to 5,000 ybp [73]. Lastly, the Mississippi River Delta (beginning at the confluence of the Red and Mississippi Rivers about 50 km north of Baton Rouge, LA) has frequently shifted channels since the LGM [70], [78]. Most relevant to the southern sample site near False River is a ∼70 km shift from the Bayou Teche channel eastward to near its current position ∼3,000 to 6,000 ybp [73], [78], [79]. Thus, the permeability of the current channel to terrestrial dispersers has likely fluctuated in the recent past due to channel switching, and this may also have produced some of the patterns predicted by meander loop cutoff. For example, if a genetic signature from these ancient channels remains, it would be expected to favor the observation of fewer migrants in control sites versus oxbow sites (as is expected under the MLC hypothesis). Channel switching however would not be expected to produce asymmetrical migration rates in the direction of the cutoff or heightened divergence within ancient meander loops (at *cis* sites). Also, for the northern locality, the observed genetic cline appears to be centered around the current channel, suggesting that, in this case, any genetic effect of the more eastern course has largely been erased. Thus, although our results here suggest a role for MLC in recent gene flow across the Mississippi River, we emphasize that MLC is only one of several ways that fluctuating river channels have compromised this dispersal barrier over the long-term.

Even if MLC is currently an important contributor to population cohesion across rivers, its long-term impacts on the structuring of populations are more dubious. The formation of the current Mississippi River meander belt system is thought to postdate the most recent glacial cycle [73], [79], being preceded by a broad web of braided streams that transported glacial loads during cycles of waning Quaternary glaciation [73], [77], [80], [81]. The population genetic effects unique to meandering river systems (e.g., MLC) thus likely arise intermittently, contingent upon the geomorphological and climatic factors that favor their formation.

The future importance of MLC to terrestrial populations is equally uncertain. Over the past century, humans have extensively modified the Mississippi River Valley (in the form of dredging, tributary alteration, and the construction of artificial cutoffs, dikes, levees, and revetments) in an attempt to create and maintain a single permanent channel [82], [83]. Thus, inasmuch as channel dynamics such as channel switching, load sharing among multiple channels, and meander migration currently contribute to the connectivity of populations and lineages on opposite sides of the river, the long-term effect of the past 100 years of engineering may be to sever this connection. Understanding the nature of riverine barrier permeability to natural populations can thus enable us to better predict the long-term consequences of river modification on organismal diversity.

## Supporting Information

Figure S1 **ML cyt** ***b*** **gene tree.**
(PDF)

Table S1 **Locality information for all sites sampled for this study.**
(DOCX)

Table S2 **Genetic divergence among sites near three Mississippi River oxbow lakes.**
(DOCX)

Table S3 **Estimates of** ***Nm*** **among pairs of sites near three oxbow lakes.**
(DOCX)

Dataset S1 **Microsatellite genotypes and GenBank accession numbers.**
(TXT)
