# Supplementary material for: Testing the Role of Meander Cutoff in Promoting Gene Flow across a Riverine Barrier in Ground Skinks (Scincella lateralis)
Source: PLoS One. 2013 May 2;8(5):e62812. doi: 10.1371/journal.pone.0062812 (PMC3642178; doi:10.1371/journal.pone.0062812)
Supplement: Table S1 — Locality information for all sites sampled for this study. (DOCX) [file pone.0062812.s002.docx]

**Table S1.** **Locality information for all sites sampled for this study.**

| **Loc #** | **County** | **State** | **N** | **Locality** | **Lat** | **Long** |
| --- | --- | --- | --- | --- | --- | --- |
| 1a | Pointe Coupee | LA | 13 | N. of Hwy 190 | 30.5546 | -91.5225 |
| 1b | Pointe Coupee | LA | 12 | Portage Canal: ~5 miles W. of False River | 30.6660 | -91.5151 |
| 2a | Pointe Coupee | LA | 18 | Chenal: off Hwy 414 | 30.6254 | -91.3777 |
| 2b | West Baton Rouge | LA | 7 | E. of Bueche Rd., just N. of W. Bueche Rd. | 30.5893 | -91.3523 |
| 3a | East Baton Rouge | LA | 18 | Hooper Road Park | 30.5327 | -91.1175 |
| 3b | East Baton Rouge | LA | 7 | Waddill WMA | 30.4905 | -91.0320 |
| 4 | Concordia | LA | 28 | Buckner Bayou: off Hwy 65, W. of L. St. John | 31.7502 | -91.4775 |
| 5 | Concordia | LA | 28 | E. of L. St. John | 31.6993 | -91.4372 |
| 6 | Adams | MS | 21 | Natchez State Park | 31.6021 | -91.1964 |
| 7 | East Carroll | LA | 29 | Bayou Macon WMA: Buck Bayou Trail | 32.8574 | -91.2895 |
| 8 | Washington | MS | 9 | W. of L. Washington | 33.0683 | -91.0691 |
| 9 | Washington | MS | 21 | Yazoo NWR | 33.0765 | -90.9732 |
| 10 | Washington | MS | 22 | Leroy Percy State Park | 33.1656 | -90.9367 |
| 11 | Montgomery | AR | 15 | Ouachita National Forest | 34.6223 | -93.8020 |
| 12 | Benton | MS | 12 | Holly Springs National Forest | 34.6994 | -89.2384 |
