# Supplementary material for: Testing the Role of Meander Cutoff in Promoting Gene Flow across a Riverine Barrier in Ground Skinks (Scincella lateralis)
Source: PLoS One. 2013 May 2;8(5):e62812. doi: 10.1371/journal.pone.0062812 (PMC3642178; doi:10.1371/journal.pone.0062812)
Supplement: Table S2 — Genetic divergence among sites near three Mississippi River oxbow lakes. (DOCX) [file pone.0062812.s003.docx]

**Table S2.** **Genetic divergence among sites near three Mississippi River oxbow lakes.**

| **Locality** | **site1** | **site2** | **D_est_** | **F_ST_** | **G'_ST_** |
| --- | --- | --- | --- | --- | --- |
| north | *trans* | control | 0.181 | 0.037 | 0.194 |
| north | *cis* | control | 0.101 | 0.014 | 0.108 |
| north | *cis* | *trans* | 0.178 | 0.030 | 0.189 |
| north | *cis1* | control | 0.220 | 0.033 | 0.231 |
| north | *cis1* | *trans* | 0.286 | 0.046 | 0.300 |
| north | *cis2* | control | 0.122 | 0.017 | 0.130 |
| north | *cis2* | *trans* | 0.193 | 0.035 | 0.207 |
| north | *cis1* | *cis2* | 0.262 | 0.036 | 0.277 |
| central | *trans* | control | 0.129 | 0.017 | 0.137 |
| central | *cis* | control | 0.169 | 0.028 | 0.182 |
| central | *cis* | *trans* | 0.177 | 0.028 | 0.188 |
| south | *trans* | control | 0.093 | 0.011 | 0.099 |
| south | *cis* | control | 0.110 | 0.013 | 0.115 |
| south | *cis* | *trans* | 0.040 | 0.008 | 0.044 |
| pure | west | east | 0.347 | 0.078 | 0.365 |

Note: for the northern locality, two *cis* sites (*cis*1 and *cis*2; see Figure 2) are considered separately and together (*cis*); all F_ST_ values are significant unless underlined.
