# Supplementary material for: Testing the Role of Meander Cutoff in Promoting Gene Flow across a Riverine Barrier in Ground Skinks (Scincella lateralis)
Source: PLoS One. 2013 May 2;8(5):e62812. doi: 10.1371/journal.pone.0062812 (PMC3642178; doi:10.1371/journal.pone.0062812)
Supplement: Table S3 — Estimates of Nm among pairs of sites near three oxbow lakes. (DOCX) [file pone.0062812.s004.docx]

**Table S3. Estimates of *Nm* among pairs of sites near three oxbow lakes.**

| **site1** | **site2** | **North** | **Central** | **South** |
| --- | --- | --- | --- | --- |
| control | *trans* | 3.059 | 2.776 | 3.911 |
| control | *cis* | 3.359 | 3.616 | 3.841 |
| *cis* | *trans* | 3.242 | 2.964 | 3.114 |
