# Supplementary material for: Testing the Role of Meander Cutoff in Promoting Gene Flow across a Riverine Barrier in Ground Skinks (Scincella lateralis)
Source: PLoS One. 2013 May 2;8(5):e62812. doi: 10.1371/journal.pone.0062812 (PMC3642178; doi:10.1371/journal.pone.0062812)
Supplement: Figure S1 — ML cyt b gene tree. (PDF) [file pone.0062812.s001.pdf]

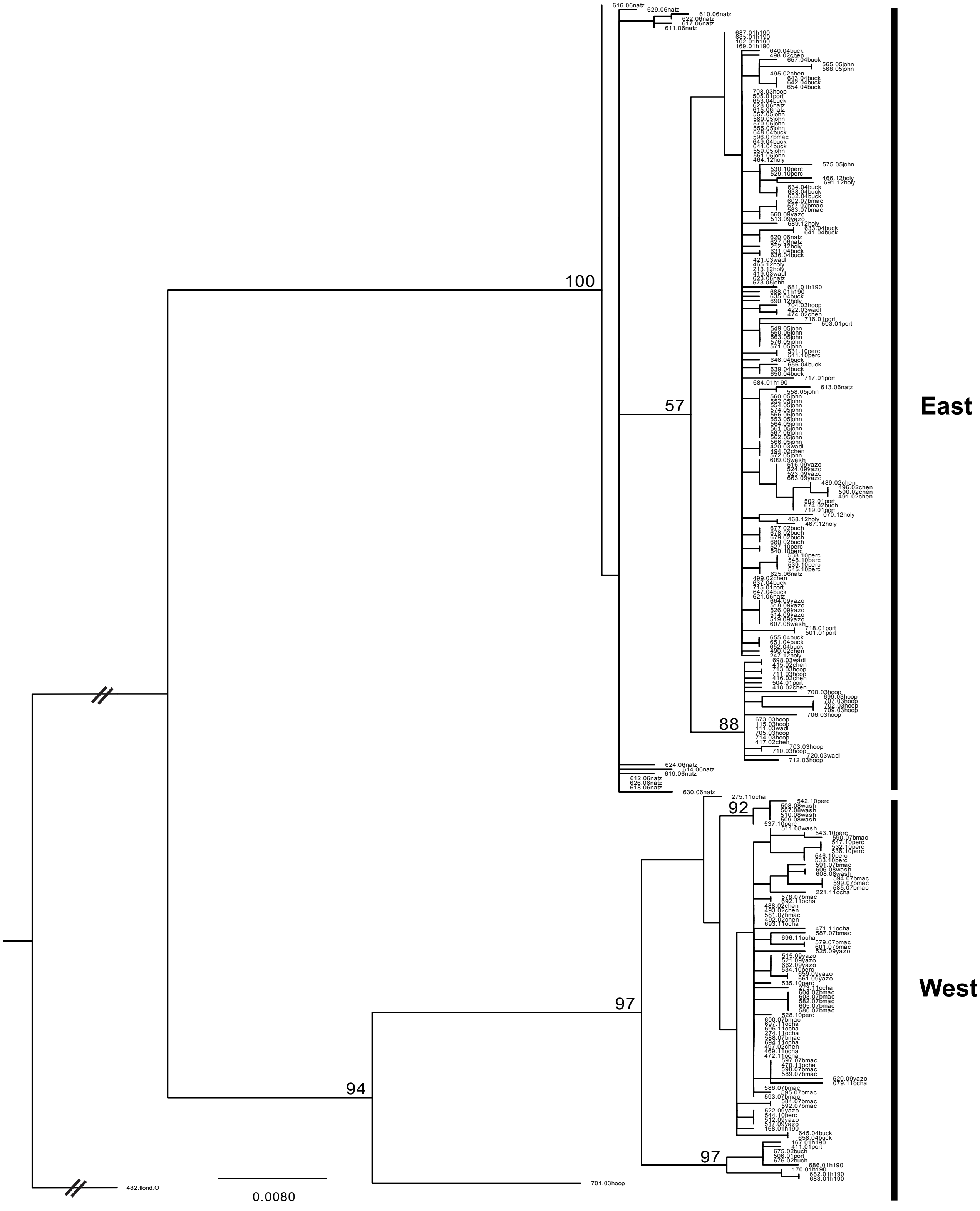

Figure S1. ML cyt*b* gene tree. This tree shows two major clades, broadly corresponding to the western or eastern side of the river. Bootstrap values are shown at select nodes and tips are labeled with a unique individual number, followed by a period, followed by a two digit locality number (matching those in Tables 1 and S1), followed by a unique locality description (matching locality descriptions in Table S1). See Dataset S1 for matching GenBank accession numbers. Hatch marks indicate shortened branches. An *S. lateralis* sample from Florida was used as an outgroup in the analysis.
